# ALDH isozymes downregulation affects cell growth, cell motility and gene expression in lung cancer cells

**DOI:** 10.1186/1476-4598-7-87

**Published:** 2008-11-24

**Authors:** Jan S Moreb, Henry V Baker, Lung-Ji Chang, Maria Amaya, M Cecilia Lopez, Blanca Ostmark, Wayne Chou

**Affiliations:** 1 Department of Medicine, University of Florida, Gainesville, Florida, USA; 2 Department of Molecular Genetics and Microbiology, University of Florida, Gainesville, Florida, USA

## Abstract

**Background:**

Aldehyde dehydrogenase isozymes ALDH1A1 and ALDH3A1 are highly expressed in non small cell lung cancer. Neither the mechanisms nor the biologic significance for such over expression have been studied.

**Methods:**

We have employed oligonucleotide microarrays to analyze changes in gene profiles in A549 lung cancer cell line in which ALDH activity was reduced by up to 95% using lentiviral mediated expression of siRNA against both isozymes (Lenti 1+3). Stringent analysis methods were used to identify gene expression patterns that are specific to the knock down of ALDH activity and significantly different in comparison to wild type A549 cells (WT) or cells similarly transduced with green fluorescent protein (GFP) siRNA.

**Results:**

We confirmed significant and specific down regulation of ALDH1A1 and ALDH3A1 in Lenti 1+3 cells and in comparison to 12 other ALDH genes detected. The results of the microarray analysis were validated by real time RT-PCR on RNA obtained from Lenti 1+3 or WT cells treated with ALDH activity inhibitors. Detailed functional analysis was performed on 101 genes that were significantly different (P < 0.001) and their expression changed by ≥ 2 folds in the Lenti 1+3 group versus the control groups. There were 75 down regulated and 26 up regulated genes. Protein binding, organ development, signal transduction, transcription, lipid metabolism, and cell migration and adhesion were among the most affected pathways.

**Conclusion:**

These molecular effects of the ALDH knock-down are associated with in vitro functional changes in the proliferation and motility of these cells and demonstrate the significance of ALDH enzymes in cell homeostasis with a potentially significant impact on the treatment of lung cancer.

## Background

Aldehyde dehydrogenases (ALDHs) are a group of NAD(P)^+^-dependent enzymes involved in the metabolism of a wide variety of aliphatic and aromatic aldehydes [1,2]. Many disparate aldehydes are ubiquitous in nature and are toxic at low levels because of their chemical reactivity. Thus levels of metabolic-intermediate aldehydes must be carefully regulated which explains the existence of several distinct ALDH families in most studied organisms with wide constitutive tissue distribution [1,2]. A systematic nomenclature scheme for the ALDH gene superfamily based on divergent evolution has been developed [3] and continues to be updated on paper [4] and on the internet by Dr. Vasilis Vasiliou and his group . According to the latest database, the human genome contains 19 ALDH functional genes and three pseudogenes [4].

The role of some of these ALDHs in endobiotic and xenobiotic metabolism has been reviewed extensively before and the specific metabolic pathways affected have been detailed [2]. Many allelic variants within the ALDH gene family have been identified, resulting in pharmacogenetic heterogeneity between individuals which, in most cases, results in distinct phenotypes [2,5] including intolerance to alcohol and increased risk of ethanol-induced cancers (ALDH2 and ALDH1A1), Sjogren-Larson Syndrome (ALDH3A1), type II hyperprolinemia (ALDH4A1), 4-hydroxybutyric aciduria (ALDH5A1), developmental delay (ALDH6A1), hyperammonemia (ALDH18A1), and late onset of Alzheimer's disease (ALDH2). Furthermore, knockouts of ALDH1A2 and ALDH1A3 in mouse are embryonic lethal and newborn lethal, respectively [6-8]. Changes in ALDH activity have been observed during experimental liver and urinary bladder carcinogenesis and in a number of human tumors [9].

One of the well studied pathways of ALDH activity is drug resistance to oxazaphosphorines. We have been interested in the role of ALDH 1A1 in drug resistance, first in hematopoietic progenitors and more recently in lung cancer. ALDH1A1, ALDH3A1, and ALDH5A1 have been shown to catalyze the oxidation of aldophosphamide [10-12]. We and others have shown that overexpression of ALDH1A1 and ALDH3A1 results in resistance to 4-hydroperoxycyclophosphamide (4-HC), an active derivative of cyclophosphamide (CP) [9-11,13,14]. More recently, ALDH3A1 was recognized as an oxidative stress response protein and thus can protect against the oxidative damage caused by other chemotherapy drugs such as etoposide [15]. We have also shown that down regulation of each enzyme by RNA antisense (AS) [16], all-trans retinoic acid (ATRA) [17] or siRNA [18] results in increased sensitivity to 4-HC.

Tetraethylthiuram disulfide (TT) (disulfiram, also known as Antabuse), an ALDH inhibitor, has been reported to affect the growth of multiple tumor cells, inhibit cancer cell invasiveness, and induce apoptosis using in vitro assays [19]. These effects were thought to be due to different mechanisms including inhibition of proteasome activity [20], increase Cu uptake with pro-oxidant effects [21,22], inhibition of NF κB [23-25], inhibition of the relaxation activity of DNA topoisomerases I and II [26], and inhibition of caspases [27].

All of the above studies indicate the biologic and clinical significance of these enzymes and, therefore, the need to better define the regulatory mechanisms involved in determining their level of expression in normal and malignant tissues. Multiple studies, mainly in animal models, have been published on the regulation of the various ALDH isozymes [28-31]. Functional genomics aim at analyzing the regulation of genes in response to physiological changes. Microarray technology revolutionized the analysis of gene expression in biological processes to enable the assessment of gene activity on a genome-wide scale. In order to be able to perform such experiment in relation to ALDH1A1 and ALDH3A1, we have aimed at achieving "knock-down" of these enzymes using siRNA approach in vitro. Indeed, we achieved > 95% "knock-down" of ALDH activity in A549 lung cancer cell line using lentiviral vectors to permanently express siRNA sequences specific to each one of the enzymes. The data presented here reveal multiple genes affected by the "knock-down" of ALDH activity which will ultimately aid in identifying the molecular events related to the activity and regulation of ALDHs expression be it in oxidative stress, response to carcinogenic aldehydes or malignant transformation.

## Methods

### Cell Lines

A549 and H522 cell lines were obtained from ATCC and maintained in -80°C freezer or cultured in RPMI 1640 medium containing 10% FBS before experiments. Cells were maintained in 5% CO2 incubator at 37°C and used for experiments in the exponential phase of their growth. These cell lines were specifically chosen for the described experiments because they are known to have high levels of ALDH1A1 and ALDH3A1 [32]. These cells were transduced with lentiviral vectors (described below) containing specific siRNA sequences against ALDH1A1 (Lenti 1 cells), ALDH3A1 (Lenti 3 cells), both vectors (Lenti 1+3 cells), and against the green fluorescent protein (GFP) gene (GFP cells, used as a control). Overall total of 5 cell lines from A549 and H522 were used throughout the experiments described here including the parent wild type cell line (WT). Microarray gene profiling was performed only on A549 cells. In order to further validate that the effects seen in the ALDH knock down cells are directly related to ALDH activity, we used known ALDH inhibitors as described below in A549 and H522 cells as well as one other lung cancer cell line, H1299, that lacks any significant expression of ALDH1A1 or ALDH3A1 [32].

### Effects of ALDH Knock-Down on Cell Growth and Motility

The assays described below were used to study the effects of ALDH knock down by siRNA on cell growth and migration as well as colony formation. Triplicates of 2 × 10^5 ^cells/ml/well were plated in 6-well tissue culture plates and grown for 72 hours. After this time, the cells were trypsinized, collected and manually counted using hematocytometer and microscope. Viability was determined by trypan blue exclusion. For these experiments, we used the five cell lines from H522 or A549. Means ± SD of growth rates were calculated and differences in growth rates were compared using the Student *t*-test.

A colorimetric assay kit (MTT based) for the non-radioactive quantification of cell proliferation and viability was purchased from Roche Diagnostics Corporation (Indianapolis, IN) and used for the cell proliferation experiments. In a 96-well plate, 1 × 10^3 ^cells/100 μL/well of each cell line were cultured for 96 hours. After the 96 hour incubation period, the labeling reagent was added to each well, followed by the solubilization solution four hours later. The cells were left overnight in the incubator, and absorbance was measured the following day using a Molecular Devices Kinetic Microplate reader at a wavelength of 570 nm as per the manufacturer's protocol. In this experiment, absorbance directly correlates with number of viable cells per well. Mean ± SD of the absorbance values were calculated for each experimental group and compared using the Student *t*-test.

For each of the cell lines, triplicates of 200 cells/ml/well in a six-well plate were cultured in RPMI-1640 + 10% FBS. The cells were allowed to grow for 5 days. The number of colonies adhered to the bottom of the plate was then counted using inverted microscope. The mean number of colonies was calculated and compared among all five cell lines originating from the A549 or H522 cell lines.

We measured the effect of down regulation of ALDH on cell migration and motility by using the in vitro scratch wound migration assay [33]. A549 monolayer confluent cells from the five different cell lines were scratched using sterile 200 μl pipette tip in 6-well plastic dishes, and after 20 hr of culture in RPMI-1640 +10% FBS, the migration ability of the cells was evaluated by the width of the wound under inverted microscope fitted with digital camera. Images of the scratch wound were taken immediately after the scratch and 20 hr later. The migration distances (in cm) of the cells were measured on printed images taken at the same magnification and the difference of the width of wounds at 0 and 20 hr was determined and the % wound healing was calculated.

### SiRNA Plasmid Constructs

**Figure 1:**
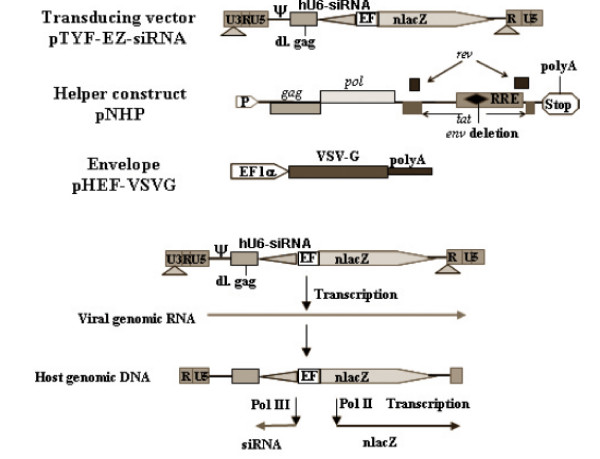
**Lentiviral constructs**. Schematic presentation of the Lentiviral constructs used in our experiments to express the various siRNA sequences against ALDH1A1, ALDH3A1, and GFP.

SiRNA constructs were made using pSilencer 2.0-U6 as vector backbone from Ambion. To generate the lentiviral siRNA constructs, the pSilencer 2.0-U6 siRNA plasmid was digested with *Pvu *II and the U6-siRNA cassette was cloned into the self-inactivating (SIN) pTYF-EFnlacZ lentiviral vector in the *Not *I site which was blunted with T4 DNA polymerase. For the constructions of ALDH LV siRNA vectors, the target site for ALDH1A1 (Genbank NM_000689) is: 5'-gtagccttcacaggatcaa-3' (nt 777–795) was chosen, and two primers were used to generate the siRNA construct; primer 1: 5'ATA CGC GGA TCC CGT AGC CTT CAC AGG ATC AAT TCA AGA G AT TGA TC-3', primer 2: 5'CGC TAG ACT AGT TAA AAA AGT AGC CTT CAC AGG ATC AAT CTC TTG AA TTG ATC -3'; the target site for ALDH3A1 (Genbank NM_000691) is: 5'-gaagatgattgcagagaca-3' (nt1167-1185), and two primers were used to generate the siRNA construct: primer 1: 5' ATA CGC GGA TCC CGA AGA TGA TTG CAG AGA CAT TCA AGA GAT GTC T-3', primer 2: 5' CGC TAG ACT AGT TAA AAA AGA AGA TGA TTG CAG AGA CAT CTC TTG AA TGT CT -3'. As a control, we similarly generated U6 promoter-driven LV-siRNA targeting GFP, as described before [34]. The lentiviral constructs with the U6-siRNA inserted in the reverse orientation were chosen for vector preparation (see Figure 1).

### Lentiviral Vector Production, Titration and Transduction

To produce lentiviral vectors, 293T cells were co-transfected with the three vector plasmids: pNHP, pHEFVSVG, and pTYF siRNA vector, and the virus supernatants were concentrated and titered as previously described [35]. For lentiviral transduction, the cells were plated at a concentration of 10^5 ^cells per well in 12-well plate to give 60–80% confluence after overnight culture and then infected at MOI of 50–100 in the presence of polybrene (8 μg/ml). Transduction efficiency was determined by the LacZ reporter gene assay as previously described [35].

After successful transduction with lentiviral constructs, we plated cells for liquid colony culture assay at 200 cells in 1 ml per 35 mm plate. On approximately day 5 of culture, we picked single colonies using inverted microscope and sterile pipette for replating and establishing clones for each of Lenti 1, Lenti 3, Lenti 1+3, and GFP cell lines. The 100% purity of the clones was validated by LacZ staining done on similarly cultured colonies from these established clones.

### ALDH Activity and Protein Measurements

In order to measure the effects of the siRNA expression, we used the spectrophotometric methods for measurement of ALDH enzymatic activity as well Western blot analysis to detect changes in the protein levels of ALDH1A1 and ALDH3A1. Western blot analysis was performed as described before [17,18]. In brief, lysates from each of the 5 cell lines mentioned above were size separated in parallel on two 12% denaturing SDS-polyacrylamide gels (BioRad, Hercules, CA), electrotransferred onto nitrocellulose membranes, blocked with 5% milk in TBS, and probed using chicken anti human ALDH1A1 and ALDH3A1 polyclonal antibodies provided generously by Dr. L. Sreerama (St Cloud University, Minneapolis, MN) and Dr. NE Sladek (University of Minnesota, Minneapolis, MN). The specificity of these antibodies has been documented by Dr. Sladek's group [36,37]. Blots were incubated with chicken anti-human ALDH1A1 and ALDH3A1 primary antibodies at 1:200 or 1:300 dilution, respectively, for 1 hour at room temperature. After washing, the secondary antibody (horseradish peroxidase-labeled rabbit anti-chicken antibody; Sigma Chemical Co., St Louis, MO) was used at 1:4000 dilution for 1 hour. Chemiluminscence method (SuperSignal, Pierce, Rockford, IL) was used for the final visualization of the protein bands on X-ray film (Super Rx, Fuji Photo Film, Tokyo, Japan). After washing and blocking, the same blots were labeled again for visualization of actin as a loading control using anti-actin antibody (Oncogene Research Products, Cambridge, MA).

The cell lysates used for Western blot analysis, were also freshly used to measure ALDH enzyme activity using the spectrophotometric assay as described before [17,18]. Briefly, the aliquots of 600 μl lysing buffer were incubated at 37°C in Beckman DLC 64 spectrophotometer cuvettes with the addition of cell lysate, 5 mM NAD^+ ^and 5 mM propionaldehyde as a substrate. The rate of change in absorbance at 340 nm was measured in 3 replicates over 5 min. A control reaction in which the substrate was not added monitored the endogenous rate of NAD^+ ^reduction. The ALDH activity was expressed in nmoles/10^7 ^cells.min.

### RNA Isolation and cDNA Synthesis

*A5*49 cell lines (WT, GFP, and Lenti 1+3) were plated in 25 cm^2 ^Corning cell culture flasks at a density of 7.5 × 10^5 ^cells/ml with RPMI 1640 medium containing 10% FBS at 37°C. Four culture sets were prepared for each experimental group. Four different clones were used for GFP and Lenti 1+3. After 24 hr incubation, media was removed from the dish and RLT buffer was added directly to the cells. Cells were scraped into this buffer and subsequently homogenized using the QIAshredder kit (Qiagen, Valencia, CA). Total RNA was then harvested using the RNeasy RNA isolation kit (Qiagen). Integrity of total RNA was validated using a Bioanalyzer (Agilent Technologies) as well as OD 260/280 ratios.

Two micrograms of total RNA were utilized for the cDNA synthesis using the cDNA synthesis kit (Affymetrix 900431) and according to the Affymetrix technical protocols. First-strand synthesis was initiated using Superscript II Reverse Transcriptase and the T7-(dt) primer (Affymetrix 900431), which contains the T7 promoter sequence followed by an oligo (dt) tract. Second strand synthesis was carried out using *Escherichia coli DNA *polymerase I, *E. coli *DNA ligase, and RNase H (Affymetrix 900431). Five microliters of purified ds cDNA was used to generate biotin-labeled cRNA using the IVT labeling kit (Affymetrix 900449). Biotin labeled cRNA was then purified from unincorporated nucleotides using the RNA cleanup kit (Affymetrix 900371). Quantity and quality of cRNA was determined by OD260/280 ratios and by using a Bioanalyzer (Agilent Technologies).

### Probe and Preparation/Staining of Array

Before using the array, biotin-labeled cRNA was fragmented for 35 minutes at 94°C. The following reagents were mixed to give the following final concentrations: fragmented cRNA (0.05 μg/ul), control oligonucleotide B2 (50 pM, Affymetrix), 20X eukaryotic hybridization controls (BioB at 1.5 pM, BioC at 5 pM, BioDn at 25 pM, and CreX at 100 pM, Affymetrix 900454), herring sperm DNA (0.1 mg/ml, Promega), acetylated BSA (50 mg/ml, GIBCO-BRL), and 2X hybridization buffer. Probe was heated to 99°C for 5 min, then transferred to 45°C for 5 min, and finally spun at maximum in a microcentrifuge for 5 min. Arrays used for this study were the Human Genome U133 Plus 2.0 Array (Affymetrix) representing over 47,000 transcripts and variants including 38,500 well-characterized human genes. Chips were interrogated with material harvested from A549 WT, Lenti 1+3 and GFP cells and each cell treatment was replicated four times using 4 chips for each experimental group, making the data from each array truly independent biological replicas. Each array was injected with 200 ul of target and rotated in a 45°C oven for 16 hours. Probe was removed from the array and the array was washed and stained using the Euk-WS2 fluidics protocol and the streptavidin-phycoerythrin (SAPE) reagent (Affymetrix).

### Microarray Scanning,, Normalization, Expression Filters, Cluster Analysis and Statistical Analysis

Scanned images (*.dat files) were analyzed with GCOS software to generate a detection call for each probe set. Hybridization single intensities between GeneChips were normalized and an expression matrix was derived with dChip [38] using the PM only model model-based expression index. Probe sets whose hybridization single intensities were never measured above background levels were removed from the data set to reduce the noise level in the data set. Probe sets whose hybridization single intensities were measured at or below background levels were identified using the Affymetrix detection call algorithm [39].

### Supervised learning, cross validation, and visualization

The modeled-based expression matrix of the probe sets passing the initial expression filter were analyzed in log space with BRB Array Tools 3.2.2 (developed by Richard Simon and Amy Peng Lam and is available online at the Biometric Research Branch of the National Cancer Institute website  to identify genes that differentiated among the treatment classes at the P < 0.001 significance threshold. The ability of gene identified at the P < 0.001 significance threshold to be differentiated among treatment classes was assessed by using leave-one-out-cross-validation (LOOCV) using a nearest-neighbor prediction model. For visualization purposes the modeled-based expression matrix of probes sets identified as significant among the treatment groups were mean centered and variance normalized using algorithms implemented in dChip.

### Functional Analysis of Transcripts

Probe sets that were significantly different at P < 0.001 and demonstrated ≥ 2 fold change in the level of gene expression were subject to comprehensive, detailed, six-level functional analysis, whereby identity was verified using GenBank accession number, aliases were compiled, and biochemical and biological functions were determined. After the identity of the probe set was confirmed, multiple internet bioinformatics and National Center for Biotechnology Information (NCBI) search engines, including PubMed searches, were used to assign the biological function. Probe sets without confirmed identities were placed in the "Unknown" category. Probe sets that could be identified but that did not have ascertainable function were classified as "Other"

### Validation of Selected Gene Expression Levels by Quantitative Real-Time RT-PCR

In order to confirm microarray data, we performed real-time RT-PCR [40] for 7 selected genes measuring the change in their level of expression. Primer/probe sets for HMOX1, MAP3K8, MGST1, CXXC5, LHX8, RAB20, STRA6 and 18S rRNA (reference control) were designed by Assays-on-Demand (Applied Biosystems, Foster City, CA, USA) and are listed in Table 1. Total RNA was obtained as described above from all five A549 cells lines described under "Cell Lines".

**Table 1:** Primers used for quantitative real time RT-PCR.

**Gene**	**Gene Product**	**Catalogue #**	**Sequence**
STRA6	Stimulated by retinoic acid Gene 6 Homolog (mouse)	Hs00223621_m1	GCACCCAGCCAAGATGGGAAAACTG

18S	Eukaryotic 18S rRNA	Hs99999901_s1	CATTGGAGGGCAAGTCTGGTGCCAG

RAB20	Member RAS oncogene family	Hs00215134_m1	ACACCGCAGGGCGGGAGCAGTTCCA

CXXC5	CXXC finger 5	Hs00212840_m1	TCCGCTGCTCTGGAGAAGGTGATGC

MAP3K8	Mitogen-activated protein Kinase 8	Hs00178297_m1	AACATGGTCATCACTCCCCAAAATG

HMOX1	Heme oxygenase 1	Hs00157965_m1	CGGCTTCAAGCTGGTGATGGCCTCC

LHX8	Zinc finger transcription factor	Hs00418293_m1	AGATCAGCTTCAGGTTATGCAAGCA

MGST1	Microsomal glutathione S-transferase 1	Hs00220393_m1	CAAGAAAGGTTTTTGCCAATCCAGA

Two micrograms of total RNA were reverse transcribed to cDNA in a 50 μl reaction containing 4 μl of 100 mM MgCl, 1.0 μl of RNase inhibitor, 4 μl of 10× PCR buffer, 1 μl of 10 mM dNTP mix, 1.0 μl of random hexamers, 1.0 μl of reverse transcriptase (Invitrogen). Mixture was incubated for 50 min at 42°C and the reaction was stopped by heating to 70°C for 15 min. For each of the Genes studied and 18S, 3 μl of 400 ng/mL diluted cDNA was added to 12.5 μl of Taqman Universal Master Mix (Applied Biosystems), 1.25 μl of 20× primer/Probe combination, and 8.25 μl of DEPC-treated water per each reaction. RT-PCR quantification and determination of expression levels were performed on ABI Prism 7900 and Sequence Detection Software 1.6 (Applied Biosystems, Foster City, CA, USA). All PCR reactions were performed in triplicates in our Gene Expression Core Facility and the results were analyzed using comparative method following normalization of expression values to 18S rRNA expression.

### The Effects of ALDH Inhibitors on ALDH Activity, Cell Growth and Gene Expression

For comparison to the siRNA induced knock-down of ALDH activity and as a way of further validation of the microarray results, we also used known inhibitors of ALDH, Tetraethylthiuram disulfide (TT, disulfiram) or diethylaminobenzaldehyde (DEAB), to study the effects of ALDH inhibition on cell growth and expression of selected genes. These inhibitors were purchased from Sigma Biochemicals (Milwaukee, WI) dissolved in DMSO and used in a final DMSO concentration of ≤ 0.1%.

In these experiments, we used the A549 and H522 parent cell lines as well as H1299 cells, a cell line known not to have any measurable ALDH activity [32]. Dose response curves (0, 0.1, 1, 10, and 100 μM) were performed for each inhibitor measuring ALDH activity, cell growth and viability. We used either manual cell counts or the calorimetric (MTT) assay, as described above. In subsequent experiments only 1 and 100 μM concentrations were used. Cells were plated at 2 × 10^5^/well in 6-well plates for 24 hr before adding the inhibitor. After 48 hr incubation with and without the inhibitors (untreated controls), the cells were harvested, counted and then used for ALDH activity assay as described above. The results were expressed as % decrease in cell proliferation or ALDH activity in comparison to untreated control.

In order to confirm that changes in the expression of selected genes are indeed specific to the siRNA induced knock-down of ALDH activity, we performed real-time RT-PCR on 3 of the 7 genes mentioned above (CXXC5, LHX8, RAB20) using RNA obtained from WT A549 cells after incubation with 5 μM of either TT or DEAB for 48 hr. Obtaining similar changes in the expression of these 3 genes after treatment with ALDH inhibitors would strongly suggest direct relationship between ALDH1A1 and ALDH3A1 activity and the changes in the gene profile demonstrated by the microarray analysis.

## Results

### ALDH Knock-Down Effect on ALDH Activity and Proteins

**Figure 2:**
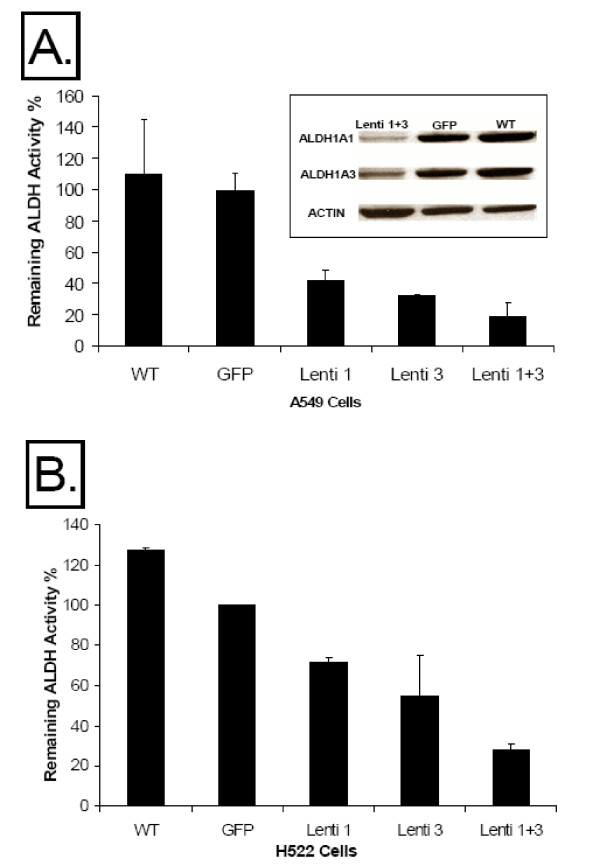
**A. The effect of siRNA against ALDH1A1 and ALDH3A1 on ALDH activity and protein**. The mean percent ± SD of remaining ALDH activity (in comparison to control GFP group) as measured by spectrophotometry enzyme activity assay is shown in A549 cells transduced by lentiviral vector containing siRNA against ALDH1A1 (Lenti 1), ALDH3A1 (Lenti 3), both vectors (Lenti 1+3), green fluorescent protein (GFP), and untreated parent cell line (WT). The results represent at least 3 different experiments. Furthermore, Western blot analysis shows the decrease in ALDH1A1 and ALDH3A1 proteins in Lenti 1+3 group in comparison to WT and GFP control groups. Actin is shown as a loading control. The results are typical of an experiment performed at least twice. **B**. Lentivirally mediated expression of siRNA constructs (shown in **A**.) results in similar effects on ALDH activity in H522 cell line.

The expression of the specific siRNAs in A549 cells resulted in a significant decrease in ALDH activity as demonstrated by spectrophotometric enzyme activity assay (Figure 2A). The effect of siRNA against each ALDH isozyme was also demonstrated by Western blot analysis (Figure 2A). With such significant decrease in ALDH activity and proteins in the Lenti 1+3 cell line, we proceeded with the microarray gene profiling experiment comparing gene expression in this cell line to that in GFP and WT control cell lines. Similar decrease in ALDH activity was obtained in H522 lung cancer cell line using the same lentiviral constructs (Figure 2B). However, we have observed the loss of siRNA effect after continuous culture of either cell line for more than 4 weeks. Because of that, fresh cells were thawed every two weeks, and ALDH activity verified before any experiments were performed.

### ALDH Knock-Down Effects on Cell Growth and Motility

**Figure 3:**
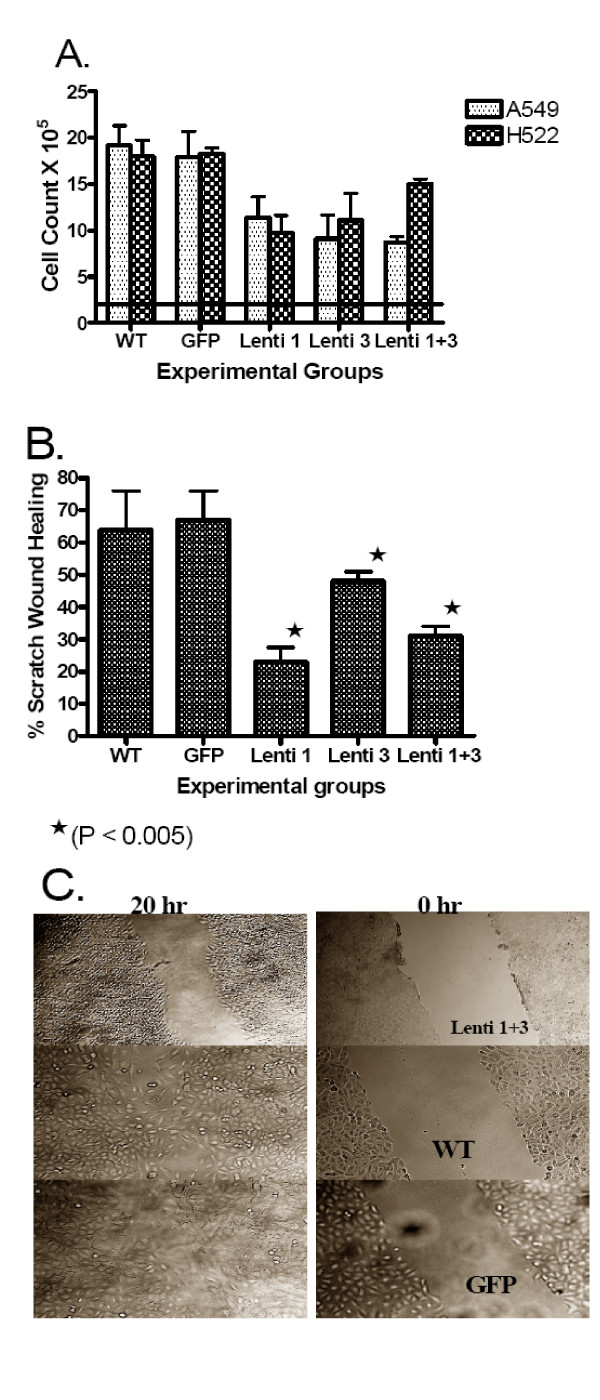
**The effect of siRNA expression on cell proliferation and motility**. **A**. Knock-down of ALDH1A1, ALDH3A1 or both results in significant decrease in cell proliferation in comparison to WT and GFP controls from A549 and H522 cells (P < 0.024, except for A549 Lenti-1 with P value of 0.052). The horizontal line indicates the starting cell number (2 × 10^5^/group) incubated, while the bars represent mean ± SD of viable cell counts after 72 hr incubation obtained from at least 2 experiments. Scratch wound migration assay results are shown. **B**. The bars represent mean ± SD of 4 measurements of wound healing in the 5 different cell lines of A549 cell. Significant decrease in cell migration into scratch wound is shown after 20 hr in cells with ALDH1A1 (Lenti-1), ALDH3A1 (Lenti-3) and both isozymes (Lenti 1+3) knock-down in comparison to WT cells or cells transduced with GFP siRNA (P < 0.005). **C**. An example of the delayed wound healing seen with the knock-down of both isozymes (Lenti 1+3 cells) on wound healing in comparison to WT and GFP cells.

A549 and H522 cells (2 × 10^5^/group) were plated for 72 hours and then counted and viability determined. Figure 3A shows the results which reflect mean ± SD of cell number from at least 4 experiments. The growth rate was significantly lower (P < 0.024) in Lenti-1, Lenti-3, and Lenti-1+3 in comparison to GFP, except for A549 Lenti-1 with P value of 0.052. No significant difference was found between the GFP and the WT. Using the MTT cell proliferation assay, we were able to show similar effects (data not shown).

A549 cells (200/petri dish in triplicates per each group) were plated for 5 days and colonies adhered to the bottom of the plates were counted after this time period. Results show that the mean number of colonies decreased significantly (range, 27–71%) for the Lenti-1, Lenti-3 and Lenti-1+3 cells in comparison to GFP and WT cells of both A549 and H522 (P < 0.016 and 0.003, respectively).

An example of the scratch wound migration test results for A549 cells are shown in Figure 3C which demonstrates delayed wound healing of the Lenti 1+3 cells in comparison to control cells. Summary of at least 4 readings are given for all the five A549 cell lines (Figure 3B). These results show significant inhibition (P < 0.005) of cell migration by knock-down of each enzyme separately as well as both enzymes (Lenti 1+3).

### Significant Changes in Gene Expression Related to "Knock-down" of ALDH Isozymes

**Figure 4:**
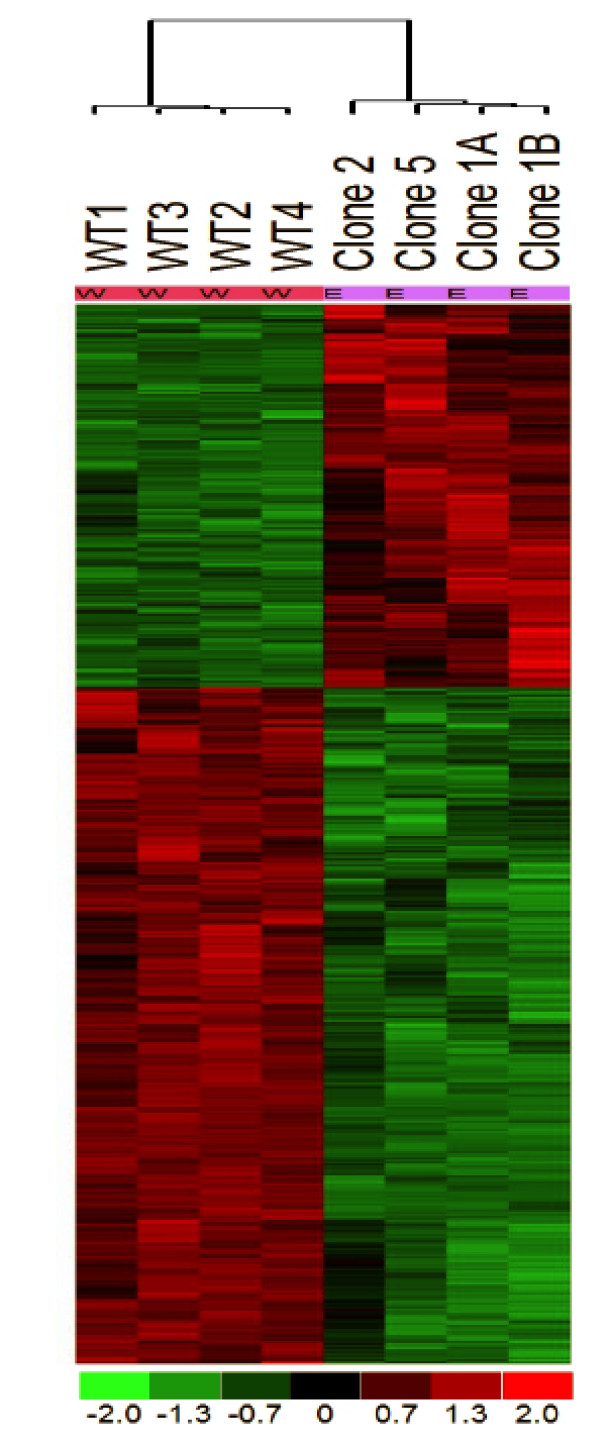
**Hybridization signal intensities varied significantly as a function of the ALDH "Knock-down"**. Four different Affymetrix U133 Plus human genome GeneChips were interrogated using labeled cRNA from WT or Lenti 1+3 cells from 4 different established clones (Vertical columns). Overall about 3735 probe sets (horizontal rows) out of total of 54675 sets were detected above background and statistically different (P < 0.001) in the Lenti 1+3 experimental group when compared to the WT group.

**Figure 5:**
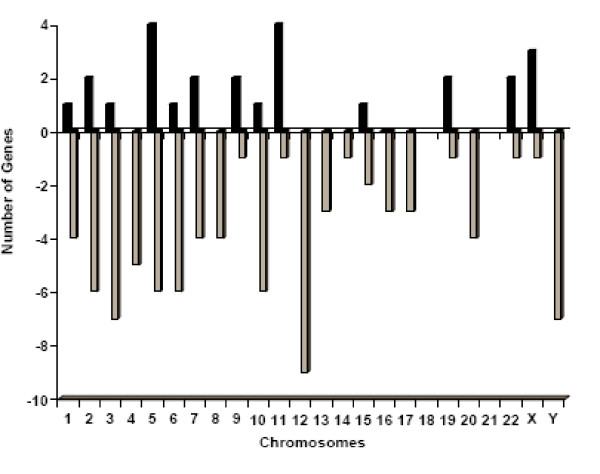
**Chromosomal clustering of affected genes**. Chromosomal clustering of 101 genes that were found to be significantly different (P < 0.001) and by ≥ 2 folds in Lenti 1+3 cells in comparison to WT and GFP control groups. The black bars represent the number of up-regulated genes, while the grey bars represent the number of down-regulated genes on each chromosome.

**Figure 6:**
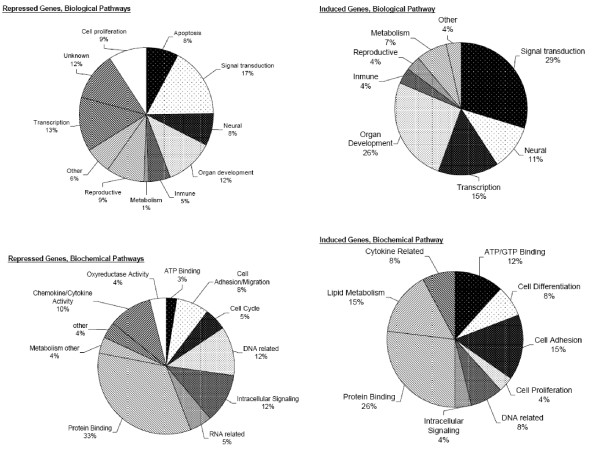
**Color-coded pies showing the distribution of induced and repressed genes into functional categories**. There were 26 significantly induced genes and 75 significantly repressed genes as a result of ALDH activity knock down in Lenti 1+3 cells compared to control groups WT and GFP. Both groups of genes were categorized once into various known biochemical pathways and again into the different biological pathways.

To construct the transcriptional profiles associated with the "knock-down" of ALDH1A1 and ALDH3A1 in A549 cells (Lenti 1+3), two other cell lines, WT and GFP, were used to interrogate the Affymetrix U133 Plus human genome GeneChips containing 38500 well-known genes. The statistical analyses are detailed above. We performed a modified F test to identify probe sets whose hybridization signal intensities varied significantly as a function of the ALDH "Knock-down". Overall about 3735 probe sets out of total of 54675 sets were detected above background and statistically different (P < 0.001) in the Lenti 1+3 experimental group when compared to the WT group (Figure 4) (supplemental material is available on  using accession number GSE8045). Many more changes in gene expression (17269 probe sets) were seen when 3 way comparisons were done among the 3 experimental groups mainly due to non-specific changes seen in the GFP group. In order to arrange the significant genes into functional framework, we chose only those genes that were significant (P < 0.001) and differed by ≥ 2 fold in the Lenti 1+3 group when compared to the WT and GFP groups. There were 26 up-regulated and 77 down-regulated genes including ALDH1A1 and ALDH3A1. We summarized the chromosomal location for these genes in Figure 5. We also researched their biological and biochemical properties using multiple internet bioinformatics search engines, web sites, and NCBI resources including BLAST, PubMed, etc. The collective results of those searches were synthesized into color-coded, mechanism-based pies of gene expression (Figure 6). These illustrations provide the relative dominance of each of the biological-biochemical processes most affected by the "Knock-down" of the ALDH isozymes. The names of these genes are tabulated in Tables 2 and 3. In summary, the affected genes are found on multiple chromosomes with the largest cluster (8 genes) observed on chromosome 12. The biological pathway most affected was signal transduction, organ development and Transcription among both the induced and repressed genes, although more pathways were found to be involved with the repressed genes. On the other hand, the biochemical pathways most affected in induced genes were protein binding, cell adhesion and lipid metabolism, while with the suppressed genes protein binding, intracellular signaling and DNA related processes were most affected (see Figure 6). As shown in Table 2, eight of the repressed genes (ID4, DDX3Y, RPS4Y1, EIF1AY, CCL20, GPC6, GPR37, and HMGA2) had the highest decrease (> 6 folds) in expression. At least of these genes, RPS4Y1 and CCL20 are highly expressed in WT A549 cells.

**Table 2:** Down regulated Genes (*P *< 0.001 and ≥ 2 folds) in Lenti 1+3 cells in comparison to WT and GFP control cells.

Gene	Fold Change in Expression	Gene	Fold Change in Expression	Gene	Fold Change in Expression
SOCS3	-2.26	KCTD12	-2.26	GPC6	-7.47
SQSTM1	-2.63	B4GALT5	-2.12	FAM33A	-2.52
WWOX	-2.67	CCNG1	-2	C6orf55	-3.05
JMY	-2.26	BCAT1	-2.43	SUSD2	-3.61
BIRC3	-3.18	ASNS	-2	LOC286144	-4.05
BCL2A1	-3.07	CXCL3	-2.82	TKT	-2.11
ITGB5	-2.53	CXCL2	-2.29	FILIP1	-5.26
TENS1	-2	SMAD1	-2.43	SMILE	-2.2
TGFBR3	-2.13	MICB	-4.35	C1orf24	-4.02
IGFBP6	-3.25	PDGFC	-2.88	CITED2	-2.01
UPK1B	-2.02	NTS	-4.77	CST1	-3.76
ANXA10	-4.03	TFAP2C	-2.35	FLJ20399	-3.63
CCL20	-9.52	SMCY	-2.79	ZFP90	-2.09
NEDD1	-2.07	DDX3Y	-7.45	UTY	-2.4
GPR37	-13.52	THRAP6	-2.48	OSRF	-2.21
PMP22	-2.11	NCOA3	-2.37	RHOBTB3	-2.02
MXI1	-2.94	USP9Y	-3.26	KIAA1571	-2.22
INHBB	-3.04	PEX13	-2.26	DRE1	-2.42
**ALDH3A1**	-3.73	VCL	-2.01	EMP1	-2.18
MAP3K1	-2	ID2	-2.03	RPS4Y1	-9.06
SCEL	-3.87	ID4	-16.12	VCL	-2.01
STEAP2	-3.6	MAFF	-5.47	EIF1AY	-18.29
TGFB2	-2.12	HMGA2	-6.69	SDPR	-3.8
**ALDH1A1**	-8.13	TFAM	-2.49		
TRAPPC2	-2	TNRC9	-3.48		
TNNT1	-3.3	MAP3K8	-3.62		
ADAM10	-2.69	FLJ23749	-2.22		

There were total of 77 genes including ALDH1A1 and ALDH3A1 (shown in bold).

**Table 3:** Up regulated genes (*P *< 0.001 and ≥ 2 folds) in Lenti 1+3 cells when compared to WT and GFP control cells.

Gene	Fold Change in Expression	Gene	Fold Change in Expression	Gene	Fold Change in Expression
TGFBR1	2.16	IL11	3.31	HMOX1	3.04
PAK3	2.51	CXXC5	2.27	CFH	2.04
PPARG	2.39	RAB20	2.11	BAAT	4.53
MGC11242	2.52	TFEC	2.25	GATM	2
FN1	2.59	OR51E2	2.22	FADS2	2.17
CNTNAP2	2.27	EHF	2.39	ANXA8	5.08
VTN	2.77	THSD2	2.52		
SULT1C1	2.05	TM4SF4	2.92		
CLDN2	3.11	FST	3.57		
PBEF1	2.21	ASRGL1	2.28		

There were total of 26 genes.

### The Overall Effect on Other ALDH Isozymes

Since the human genome contains 19 ALDH functional genes and three pseudogenes [5], we sought to analyze the effect of the siRNA sequences used here on the other members of the ALDH family. We interrogated the data set of probes that showed changes in gene expression and pulled out the ALDH genes and compared changes among the 3 different experimental groups. The data is summarized in Table 4, and it shows that the siRNA sequences used are quite specific which validates our data further. ALDH1A1 and ALDH3A1 transcripts were decreased by about 7 and 4 folds, respectively. Total of another 12 ALDH genes showed some expression variability, but it was either non-specific (similar in GFP and Lenti 1+3) or minimal.

**Table 4:** Changes in the expression of the different ALDH genes profiled in the microarray including ALDH1A1 and ALDH3A1.

Gene Symbol	Accession No.	Mean of Intensities		
		Lenti 1 + 3	GFP	WT
**ALDH3A1**	NM_000691	900.2	2300.5	3363.7
**ALDH1A1**	NM_000689	1366.7	9197.6	9390.9
ALDH2	NM_000690	2172.6	828	3083.2
ALDH3B1	NM_000694	619.7	349.4	617.1
ALDH3B2	NM_000695	81.2	99.5	113.6
ALDH1A3	NM_000693	94.5	229.3	144.5
ALDH1A2	AB015228	81.1	125.9	125.1
ALDH9A1	NM_000696	693.2	563.8	809.5
ALDH18A1	U76542	807.3	554.4	549
ALDH1L1	NM_012190	120.6	74	108.4
ALDH7A1	BC002515	450.4	395	542.1
ALDH5A1	NM_001080	139.1	72.5	112.2
ALDH4A1	NM_003748	231.9	188.1	265.4
ALDH6A1	AW612403	77	40.2	74.5

Note: ALDH3B1, ALDH3B2, ALDH4A1, ALDH6A1, and ALDH18A1 had two sets of probes, which demonstrated similar changes as shown in the table above.

### Comparison of Apparent Gene Expression among DNA Array and Real-Time RT PCR

**Figure 7:**
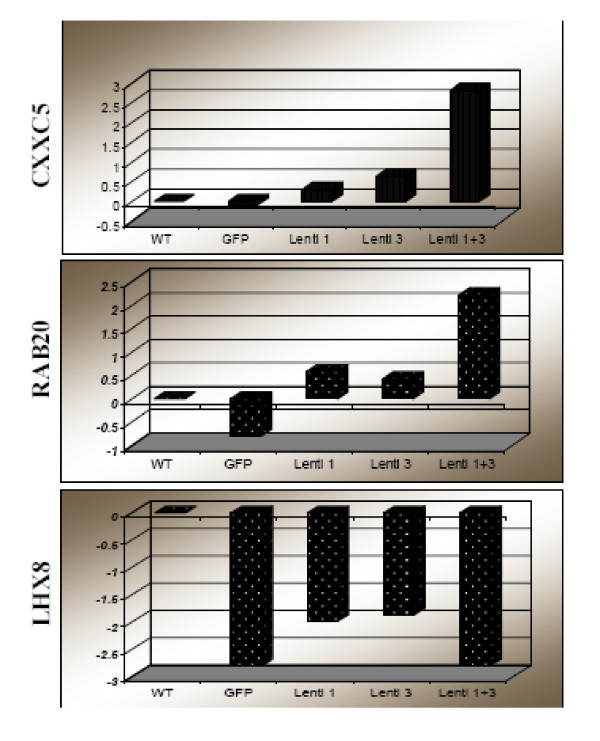
**Quantitative real-time RT-PCR to validate the results of microarray analysis**. Results of quantitative real-time RT-PCR for 3 (CXXC5, RAB20, LHX8) out of 7 seven genes studied are shown here. These genes were found to be significantly different (P > 0.001) by microarray analysis in Lenti 1+3 experimental group, in which both ALDH1A1 and ALDH3A1 were knocked down by siRNA expression, in comparison to WT control group. The real-time RT-PCR was performed on RNA obtained from 5 cell lines including cells that express siRNA against ALDH1A1 only (Lenti 1), against ALDH3A1 only (Lenti 3), or against green fluorescent protein (GFP) as another control, in addition to the Lenti 1+3 and WT groups. The results are expressed as the log 10 of relative change in each gene expression with the denominator being the 18S as a reference control and using the fold increase (+) or decrease (-) compared to the WT/18S ratio as the baseline expression value. The direction (up- or down-regulation) for all 7 genes confirmed the results of microarray analysis even in the Lenti 1 and Lenti 3 cells. LHX8 was the only gene out of the 7 tested that was non-specifically down-regulated since it showed similar changes in the GFP control group as well.

In order to assess the validity of the microarray results, we used real-time RT-PCR to compare the expression of 8 transcripts with significant expression changes (P < 0.001 and ≥ 2 folds in Lenti 1+3) per the microarray in all 5 cell lines mentioned in Figure 2A include WT, GFP, Lenti 1, Lenti 3 and Lenti 1+3. Figure 7 shows results for 3 genes CXXC5, RAB20, and LHX8 that confirm the results seen in the 3 experimental groups of the microarray. The increase in CXXC5 and RAB20 expression was synergistic with the knock-down of both ALDH1A1 and ALDH3A1, while the effect seen on LHX8 seems to be related to the lentiviral vector integration/effect because it is seen equally in all cell lines except WT. HMOX1 and MGST1 were increased in Lenti 1+3 and to a lesser degree with either Lenti 1 or Lenti 3. On the other hand, MAP3K8 expression was decreased in Lenti 1+3 only, while STRA6 was decreased in Lenti 1+3 and Lenti 1. Thus, the real-time RT PCR measurements of all 7 transcripts showed agreement of 100% with the microarray results. LHX8 was the only gene out of this group exhibiting non specific change in expression, both by microarray as well real-time RT PCR. On the other hand, MGST1 and STRA6 change in expression did not meet the cutoff of ≥ 2 folds.

### ALDH Inhibitors Effects on Cell Proliferation and Gene Expression

**Figure 8:**
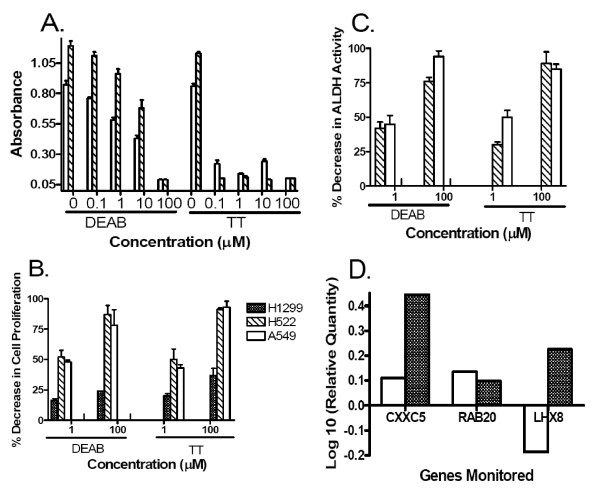
**The effects of ALDH inhibitors DEAB and TT on ALDH activity, cell proliferation and gene expression**. **A**. Dose-response effect of DEAB and TT on cell proliferation of A549 and H522 lung cancer cell lines. A colorimetric non-radioactive (MTT based) assay was used as described in Methods to quantify cell proliferation. The results are expressed as the mean ± SD of absorbance and reflect 8 measurements from 2 experiments. Overall, significant decrease (P < 0.023) in cell proliferation was seen with both inhibitors. **B **&**C**. H1299 lung cancer cell line with no measurable ALDH activity and A549 and H522 cells were cultured with or without ALDH inhibitors and cell proliferation and ALDH activity were measured as described in Methods. The results are expressed as % decrease (mean ± SD of at least 2 experiments) in cell proliferation (B) or ALDH activity (C) in comparison to untreated controls. The results demonstrate that the effect of ALDH inhibitors (1 and 100 μM) on H1299 cell proliferation is significantly (P < 0.029) less than that on A549 and H522. These results suggest that the effect on cell proliferation is mostly due to the inhibition of ALDH activity. **D**. After 48 hr incubation of A549 cells with 5 μM of DEAB (dotted bars) or TT (clear bars), RNA was extracted and real-time RT-PCR was performed, as described in Methods, for the 3 genes shown in Figure 7. The results are expressed as the log 10 of relative change in gene expression when compared to untreated WT cells (see Figure 7 legend). These results confirm that the increase in CXXC5 and RAB20 expression is most likely related to the decrease in ALDH activity. The effect on LHX8 was different by the two inhibitors and therefore non specific to ALDH activity inhibition.

To further validate our results, cells (1000/well) were incubated with ALDH inhibitors, DEAB or TT, for 72 hrs and cell proliferation measured by the calorimetric MTT based assay (Figure 8A). Absorbance was significantly lower (P < 0.0001) after adding any concentration of TT to A549 cells as well as to H522 cells. Absorbance was significantly lower (P < 0.023) after adding 0.1 μM DEAB to A549 cells, while for H522 cells, the absorbance was significantly lower (P < 0.007) only after adding ≥ 1 μM of DEAB. The slight recovery of cell proliferation at 10 μM concentration of TT has been described before [19]. These proliferation inhibitory effects seem to be specific to the inhibition of ALDH activity since the use of these inhibitors in H1299 cells lacking ALDH1A1 and ALDH3A1 expression or any significant ALDH activity [32] resulted in significantly less effects (P < 0.029) of either TT or DEAB on H1299 cell growth in comparison to H522 and A549 cell lines (Figure 8B). The dose-response effect of both inhibitors (1 and 100 μM) on ALDH activity in A549 and H522 cells is shown in Figure 8C.

Furthermore, 3 of the genes (CXXC5, RAB20 and LHX8, shown in Figure 7) were examined using real-time RT-PCR after 48 hr incubation of A549 cells with 5 μM DEAB or TT. The results show similar changes in the expression of CXXC5 and RAB20 to what was seen in the microarray results and real-time RT-PCR (Figure 8D). Once again change in LHX8 expression was affected differently by the two different inhibitors, which is suggestive of non-specific effect.

## Discussion

In this study, we have used microarray analysis to assess global changes in gene expression that result from the knock-down of ALDH1A1 and ALDH3A1 in lung cancer cell line in order to gain insight into the underlying mechanisms that lead to up regulation of these two isozymes in some cancers. The goal of this study was also to identify genes or gene networks that interact with these isozymes and may be responsible for their biological effects. We have been successful in achieving significant knock-down of these two isozymes that resulted in abolishing up to 95% of ALDH activity in A549 cells used in these experiments. We have seen significant changes in the rate of growth of H522 and A549 cell lines as well as significant decrease in cell motility and migration of A549 cells with the knock-down of ALDH1A1 and/or ALDH3A1. Similar effects have been described before with the in vitro use of low doses of disulfiram (TT) [41,19], and we were able to show similar effects on cell growth by the addition of DEAB or TT, both are known ALDH inhibitors, to cell cultures in experiments presented above. As we mentioned in the introduction, multiple other mechanisms have been elucidated for the TT effects on cell growth and apoptosis, however our results suggest that it may have to do mostly with ALDH activity either directly or indirectly through the multiple metabolic effects of ALDH isozymes. This is mainly based on two facts that are reported here for the first time: another ALDH inhibitor such as DEAB causes similar effects on cell growth like TT and the microarray gene analysis presented here reveals that multiple genes are affected with the knock-down of ALDH1A1 and ALDH3A1.

Our results also show that the siRNA sequences used here are specific to these two isozymes and do not significantly affect 12 other ALDH genes according to the microarray analysis. Thus, we feel confident that the findings presented here are indeed true representation of the changes in ALDH1A1 and ALDH3A1 activity.

The role of ALDH isozymes in many endobiotic and xenobiotic metabolic pathways has been described in details before. ALDH1A1 and/or ALDH3A1 have been implicated in retinoic acid metabolism, metabolism of aldehydes produced during lipid metabolism, alcohol metabolism, cyclophosphamide metabolism, and oxidative stress response [5]. We have reported the upregualtion of ALDH1A1 mRNA and protein in hematopoietic progenitors by interleukin-1 and tumor necrosis factor alpha [42]. Furthermore, some mutations in ALDH isozymes have been described that lead to specific clinical phenotypes or diseases, and many of these diseases are characterized by neurologic abnormalities [5]. Disulfiram (Antabuse, TT), an ALDH inhibitor used to treat alcoholism, has been reported to cause significant neurologic toxicity in patients [43,44]. Thus, it is not surprising to detect such wide spread changes in the levels of multiple genes as revealed in our microarray analysis. These include down and up regulated genes (≥ 2 fold) that are involved in neural biological pathways, such as GPR37, PMP22, NTS, KCTD12 (PFET1), SMAD1 (all down regulated ≥ 2 fold), as well as PAK3, CNTNAP2, and OR51E2 (up regulated ≥ 2 fold). Furthermore, several biochemical pathways are affected in the A549 cells expressing siRNA against both ALDH1A1 and ALDH3A1 such as protein binding, cell adhesion, intracellular signaling, and lipid metabolism. These pathways are inter-related since protein binding could be in the center of intracellular signaling; while lipid metabolism could lead to changes in membrane structure and, as a result, changes in adhesion properties.

The most important data we hoped to obtain from the microarray analysis was identifying genes (transcription factors) that may have a role in the regulation of expression of these two enzymes and may give clues to the mechanisms involved in their up regulation in the different types of cancer. Previous studies have shown that ALDH1A1 can be up-regulated by HOX11 [45,46], and that peroxisome proliferator activated receptor gamma (PPAR-γ) affect the expression of ALDH3A1 via other unknown transcription factors [28]. Furthermore, the involvement of the Ah receptor nuclear translocator (ARNT) in xenobiotic induction of ALDH3A1 has been established [47]. Our results confirm the involvement of PPAR-γ in ALDH regulation which was found to be significantly up regulated by ALDH knock down (Table 3). Also in this study, we have identified significant changes in some transcription factors such as TFEC which was up regulated (≥ 2 folds), and TFAM, LISCH7, HBP1 and HOXA5 which were down regulated (≥ 2 folds). HBP1 is highly expressed in bronchial epithelium [48], while HOXA5 was reported to be abnormally expressed in NSCLC [49]. Again, these observations will open the door for further studies in order to define mechanisms involved in the regulation of ALDH1A1 and ALDH3A1.

Most importantly, our finding showing significant decrease in the levels of 8 genes by > 6 folds implies the importance of ALDH activity in cell homeostasis and cancer transformation. These include CCL20, GPR37, DDX3Y, ID4, GPC6, RPS4Y1, EIF1AY, and HMGA2. According to the microarray results, RPS4Y1 and CCL20 are highly expressed in A549 cells, while the other 5 genes are expressed at lower levels. Information about the biological functions of these 8 genes was extracted form reviewing relevant publications and is summarized in Table 5. Some of these genes are involved in transcription regulation, cell growth, differentiation and apoptosis [50-56]. Furthermore, seven of these genes (not including RPS4Y1) were implicated and studied in various cancers, but only HMGA2 was recently reported to be over expressed in lung cancer and inversely associated with survival [56]. HMGA2 is found on chromosome 12q13-15, and indeed clinically relevant chromosome 12 abnormalities have been reported among the frequent chromosomal abnormalities described in non-small cell lung cancer [57-59]. Furthermore, our data show that the largest cluster of genes affected by ALDH knock-down was on chromosome 12. Furthermore, 3 of the highly affected genes are located on Y chromosome (Table 5). Deletion of Y chromosome is one of the frequent abnormalities reported in NSCLC [60-63] and is associated with malignant transformation and development of lung cancer [64-66]. We have recently reported our immunohistochemistry results on archived pathological specimens of patients with lung cancer which showed that both isozymes are highly expressed in non-small cell primary lung cancers [67]. Overall, these findings indicate that some of the genes identified in this study are good candidates for future investigation aiming at defining gene networks in which ALDH isozymes play an important role in cancer biology and may, in turn, enhance our knowledge of overall disease process.

**Table 5:** Genes that were highly affected by the knock-down of ALDH1A1 and ALDH3A1 in A549 lung cancer cell line.

**Symbol of Gene**	**GB Accession Number**	**Chromosome Location**	**Fold Change in Expression**	**Known Function**
CCL20 (MIP-3α)	NM_004591	2q33-q37	- 9.5	Chemokine ligand 20 is a ligand for CCR6. Upregulated in hepatocellular carcinoma. Part of the immune and inflammatory response in the lungs. Lack of expression of CCR6 on Lewis lung cancer decreases metastasis. Important for invasiveness of pancreatic and colon cancers. Expressed in oral SCCA.

GPR37	U87460	7q31	- 13.5	Highly expressed in brain neurons. If parkin absent, it accumulates and causes cell death & degeneration (Parkinson's disease). The neuropeptide head activator (HA) is a high-affinity ligand for GPR37. Hypermethylated frequently in AML.

DDX3Y	NM_004660	Yq11	- 7.45	Belongs to the DEAD-box RNA helicase family. Alter RNA secondary structure. Role in RNA splicing and transport. A candidate for tumor suppressor gene.

ID4	AW157094	6p22-p21	-16.12	Transcription factor. It inhibits binding to DNA and transcriptional transactivation by heterodimerization with bHLH protein. Expressed in small cell lung cancer. It is deregulated in ALL with t(6;14), gastric adenoca, and bladder cancer. Part of TGF-β pathway. Possible tumor suppressor gene

HMGA2	NM_003483	12q15	- 6.6	Belongs to non-histone chromosomal high mobility group protein family. It affects chromatin structure & DNA binding. Involved in obesity, pituitary adenomas, lipomas, serous cancer of overy, MDS, malignant transformation of oral cancer. Overexpressed in NSCLC, thyroid cancer, CML, prostate and possibly others. Detection of its mRNA in blood, prognostic for breast cancer patients.

GPC6	AI651255	13q32	- 7.5	A cell surface proteoglycan. Involved in cell growth and division. Co-receptor for growth factors

RPS4Y1	NM_001008	Yp11.3	- 9.06	Ribosomal protein 4 belongs to the S4E family. Involved in translation

EIF1AY	NM_004681	Yq11.222	-18	Eukaryotic initiation factor 1A, required for the binding of 43S (a 40S subunit) to the 5' end of capped RNA.

## Conclusion

We have been able to show that reduction in ALDH activity by siRNA or ALDH inhibitors may have significant effects on cell growth and proliferation possibly through effects on wide spectrum of genes with different biological roles in lung cancer cells. We predict that these results will have significant implications on defining the biological significance and mechanisms involved in high expression of ALDH isozymes in NSCLC, and therefore, impact the treatment of lung cancer.

## List of abbreviations

ALDH: aldehdye dehydrogenase; ALDH1A1 and ALDH3A1: aldehyde dehydrogenase class 1A1 and 3A1; DEAB: diethylaminobezaldehyde; TT: tetraethylthiuram disulfide or disulfiram; NSCLC: non small cell lung cancer; Lenti 1 or Lenti 3: cells expressing siRNA against ALDH1A1 or ALDH3A1 using lentivirus; Lenti 1+3: cells expressing siRNA against ALDH1A1 and ALDH3A1; WT: refers to wild type cells; GFP: refers to cells expressing siRNA against the green fluorescence protein.

## Competing interests

The authors declare that they have no competing interests.

## Authors' contributions

JSM was behind the hypothesis tested here, designed and analyzed the studies, his laboratory (MA and BO) performed the cell transduction, RNA extraction and cell culture experiments; WC prepared the lentiviral constructs with the specific siRNA in Dr Chang's Laboratory; HVB and his laboratory technician (MCL) performed the microarray hypridization and analysis. All authors read and approved the final manuscript.
